# A Brain-Machine Interface Based on ERD/ERS for an Upper-Limb Exoskeleton Control

**DOI:** 10.3390/s16122050

**Published:** 2016-12-02

**Authors:** Zhichuan Tang, Shouqian Sun, Sanyuan Zhang, Yumiao Chen, Chao Li, Shi Chen

**Affiliations:** 1 Industrial Design Institute, Zhejiang University of Technology, Hangzhou 310023, China; 2 College of Computer Science and Technology, Zhejiang University, Hangzhou 310027, China; ssq@zju.edu.cn (S.S.); syzhang@cs.zju.edu.cn (S.Z.); superli@zju.edu.cn (C.L.); shelleych@zju.edu.cn (S.C.); 3 Fashion Art Design Institute, Donghua University, Shanghai 200051, China; cym@mail.dhu.edu.cn

**Keywords:** BMI, ERD, ERS, upper-limb exoskeleton, motor imagery, motor execution

## Abstract

To recognize the user’s motion intention, brain-machine interfaces (BMI) usually decode movements from cortical activity to control exoskeletons and neuroprostheses for daily activities. The aim of this paper is to investigate whether self-induced variations of the electroencephalogram (EEG) can be useful as control signals for an upper-limb exoskeleton developed by us. A BMI based on event-related desynchronization/synchronization (ERD/ERS) is proposed. In the decoder-training phase, we investigate the offline classification performance of left versus right hand and left hand versus both feet by using motor execution (ME) or motor imagery (MI). The results indicate that the accuracies of ME sessions are higher than those of MI sessions, and left hand versus both feet paradigm achieves a better classification performance, which would be used in the online-control phase. In the online-control phase, the trained decoder is tested in two scenarios (wearing or without wearing the exoskeleton). The MI and ME sessions wearing the exoskeleton achieve mean classification accuracy of 84.29% ± 2.11% and 87.37% ± 3.06%, respectively. The present study demonstrates that the proposed BMI is effective to control the upper-limb exoskeleton, and provides a practical method by non-invasive EEG signal associated with human natural behavior for clinical applications.

## 1. Background

The upper-limb exoskeleton is designed with the goal of restoring functions and assisting activities of daily living (ADL) to those elderly, disabled and injured individuals [1,2,3,4]. Electromyography (EMG) and force sensors are two of the widely used control methods to control those exoskeletons based on the user’s motion intention [5,6,7,8,9,10,11,12]. However, for the seriously amputated and paralyzed people who cannot generate sufficient muscle signals or movements, they are not able to provide the completed EMG and force signals, which will affect the estimation of motion intention. The brain-machine interfaces (BMI) based on electroencephalogram (EEG) have received huge interest due to their potential [13]. A non-invasive recording procedure is safer and easy to apply, and it is potentially applicable to almost all people including those seriously amputated and paralyzed.

For many years, scholars have found that motor execution (ME) and motor imagery (MI) can change the neuronal activity in the primary sensorimotor areas [14,15,16]. When humans execute or imagine the movement of unilateral limb, the power of mu and beta rhythms will decrease or increase in the sensorimotor area of the contralateral hemisphere and the ipsilateral hemisphere, respectively [17]. The former case is called event-related desynchronization (ERD), and the latter event-related synchronization (ERS) [18,19]. The ERD/ERS patterns can be utilized as important features in the discrimination between right hand and left hand movement, and hand and foot movement [17]. In the frequency bands varying between 9 and 14 Hz and between 18 and 26 Hz of EEG signals, the ERD/ERS patterns can provide best discrimination between left and right hand movement imagination, and the accuracy of online classification is more than 80% [20]. To discriminate between hand and foot movement, the online classification accuracy is even as high as 93% [21]. More recently, researchers have focused on using ERD/ERS patterns to control the EEG-based BMI applications [22,23,24]. Ang et al. [25] classified the ERD/ERS patterns as “go” and “rest” using the Common Spatial Pattern algorithm to trigger a 2 degree-of-freedom MIT-Manus robot developed by an MIT research group for reaching tasks. Sarac et al. [26] presented a systematic approach that enables online modification/adaptation of robot assisted rehabilitation exercises by continuously monitoring intention levels of patients utilizing an EEG-based BCI. Linear Discriminant Analysis (LDA) was used to classify ERS/ERD patterns associated with MI. Pfurtscheller et al. [27] used brain oscillations (ERS) to control an electrical driven hand orthosis (open or close) for restoring the hand grasp function. The subjects imagined left versus right hand movement, left and right hand versus no specific imagination, and both feet versus right hand, and achieved an average classification accuracy of approximately 65%, 75% and 95%, respectively. Bai et al. [28] proposed a new ERD/ERS-based BCI method to control a two-dimensional cursor movement, and found the online classifications of the EEG activity with ME/MI were: >90%/∼80% for healthy volunteers and >80%/∼80% for the stroke patient. These studies mainly show the feasibility of BMI integrated robots and orthosis. However, few studies have focused on the upper-limb exoskeleton control using ERD/ERS patterns.

This paper aims to investigate whether self-induced variations of the EEG can be useful as control signals for an upper-limb exoskeleton developed by us. We proposed a BMI based on ERD/ERS, and tested this approach in one closed-loop experiment of increasing real-life applicability. The experiment consists of two phases: the decoder-training phase and the online-control phase. In the decoder-training phase, subjects performed ME or MI under two paradigms, i.e., left versus right hand movement and left hand versus both feet movement. We investigated nine classification strategies (three classifiers × three train–test ratios) in order to select the best classifier and train–test ratios resulting in a best classification performance. Then, based on the best classification strategy, the paradigm achieving a higher classification accuracy was chosen to the online-control phase. In the online-control phase, the trained decoder was tested in two scenarios with visual feedback. In the first scenario, subjects without wearing the exoskeleton (it was hung up beside the subject) controlled it by using ME or MI; in the second scenario, subjects wearing the exoskeleton on the right arm controlled it by using ME or MI.

## 2. Method

### 2.1. Subjects and Data Acquisition

Four able-bodied subjects (three males and one female, age range: 27–31 years old) participated in this study. They were all right handed according to the Edinburgh inventory [29], and they all had a good MI ability according to the testing method from [30]. The experiment was carried out in accordance with the approved guidelines. The experimental protocol was reviewed and approved by the human ethical clearance committee of Zhejiang University. Informed written consent was obtained from all subjects that volunteered to perform this experiment.

EEG data were recorded from 28 active electrodes (FC5, FC3, FC1, FCz, FC2, FC4, FC6, C5, C3, C1, Cz, C2, C4, C6, CP5, CP3, CP1, CPz, CP2, CP4, CP6, P5, P3, P1, Pz, P2, P4, P6) attached on an EEG cap in conjunction with the ActiveTwo 64-channel EEG system (BioSemi B.V., Amsterdam, The Netherlands). This system replaces the ground electrodes in conventional systems with two separate electrodes (CMS and DRL) [31]. The reference electrode was placed on the left mastoid. EEG was digitized at 512 Hz, power-line notch filtered at 50 Hz, and band-pass filtered at between 0.5 and 100 Hz. Before electrode attachment, alcohol was used to clean the skin, and conductive gel was used to reduce electrode resistance.

### 2.2. Experiment Procedure

The experiment consists of two phases: the decoder-training phase and the online-control phase. During recording, a quiet environment with dim light was provided to maintain the subjects’ attention level. They were asked to keep all muscles relaxed; in addition, they were also instructed to avoid eye movements, blinks, body adjustments, swallowing or other movements during the visual cue onset. Cues were presented on a computer screen, and occupied about $1.2^{\circ }$ of the visual angle.

#### 2.2.1. Decoder-Training Phase

**Figure 1:**
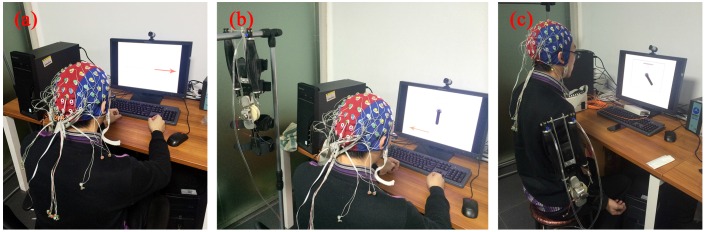
Experimental setup. (**a**) in the decoder-training phase, subjects were instructed to perform ME or MI under two paradigms, i.e., left versus right hand movement and left hand versus both feet movement. The left arrow indicated left wrist extension, and the right arrow indicated right wrist extension for Sessions 1 and 2 or both feet dorsiflexion for Sessions 3 and 4. (**b**,**c**) in the online-control phase, the trained decoder using the selected paradigm was tested in two scenarios with a visual feedback. In the first scenario, subjects without wearing the exoskeleton (it was hung up beside the subject) controlled it by using ME or MI; in the second scenario, subjects wearing the exoskeleton on the right arm controlled it by using ME or MI.

**Figure 2:**
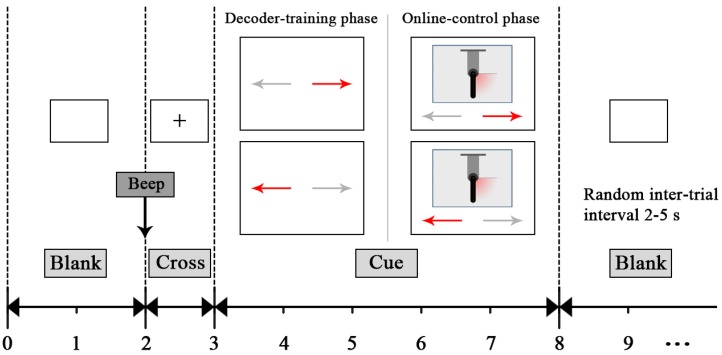
Timing of one trial used in the experiment. Each trial started with the presentation of an acoustical warning tone and a cross (second 2). One second later, a cue was randomly chosen in one of two (“←” and “→”), which indicated the movement to be executed or imagined. This cue was presented visually by means of a left/right arrow, which appeared in the middle of the computer screen. Subjects had to perform the ME and MI for five seconds, until the screen content was erased (second 8). After a short pause (random duration between two and five seconds), the next trial started.

During the decoder-training phase, subjects were seated in a comfortable chair with their forearms semi-extended on the desk, as shown in Figure 1a. Subjects were instructed to perform ME or MI under two paradigms, i.e., left versus right hand movement and left hand versus both feet movement. Each subject completed 480 trials in total, consisting of random sequences of 120 trials of four sessions (see Table 1). As shown in Figure 2, each trial started with the presentation of an acoustical warning tone and a cross (second 2). One second later, a cue was randomly chosen in one of two (“←” and “→”), which indicated the movement to be executed or imagined: left wrist extension, and right wrist extension for Sessions 1 and 2 or both feet dorsiflexion for Sessions 3 and 4, respectively. This cue was presented visually by means of a left/right arrow, which appeared in the middle of the computer screen. Subjects had to perform the ME and MI for 5 s, until the screen content was erased (second 8). After a short pause (random duration between 2 s and 5 s), the next trial started. We asked the subjects to relax their muscles and pay attention to the visual cue. We investigated the two paradigms and chose the one achieving a better classification performance for the online-control phase.

#### 2.2.2. Online-Control Phase

**Figure 3:**
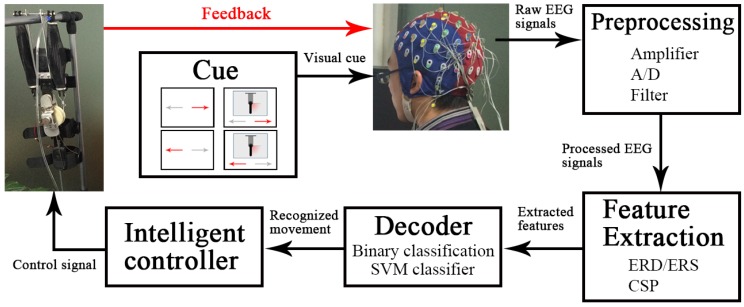
Control strategy of the upper-limb exoskeleton. Subjects performed ME or MI according to the cue. The presentation of cue was same as in the decoder-training phase. After the preprocessing and feature extraction for the raw EEG signals, the extracted features were input into the trained decoder and were classified to recognize which movement the subjects were executing or imagining. Depending on the classification result, the intelligent controller sent control signals to the exoskeleton to make it perform the corresponding action. Feedback was given by a vertical bar simulating the active part of the exoskeleton on the computer screen (Scenarios 1 and 2) or by the exoskeleton mounted on the right arm (Scenario 2). The feedback bar moved the same angle as the exoskeleton in real time.

During the online-control phase, the trained decoder using the selected paradigm was tested in two scenarios. In the first scenario, subjects without wearing the exoskeleton (it was hung up beside the subject) were seated in a comfortable chair with their forearms semi-extended on the desk, as shown in Figure 1b. In the second scenario, subjects wearing the exoskeleton on the right arm were seated in a comfortable chair with their left forearm semi-extended on the desk and right arm hanging vertically to the sagittal plane, as shown in Figure 1c. The upper-limb exoskeleton with one degree of freedom could perform two actions: flexion and extension. It was used to assist the elbow movement for users in daily activities. In two scenarios, the exoskeleton could move by a binary control while subjects performed ME or MI under the selected paradigm. The control strategy is shown in Figure 3. Subjects performed ME or MI according to the cue. The presentation of the cue was the same as in the decoder-training phase. After the preprocessing and feature extraction for the raw EEG signals, the extracted features were input to the trained decoder and were classified to recognize which movement the subjects were executing or imagining. Depending on the classification result, the intelligent controller sent control signals to the exoskeleton to make it perform the corresponding action. Feedback was given by a vertical bar simulating the active part of the exoskeleton on the computer screen (Scenarios 1 and 2) or by the exoskeleton mounted on the right arm (Scenario 2). The feedback bar moved the same angle as the exoskeleton in real time. There were two sessions in each scenario, and each session consisted of 60 exoskeleton actions.

During the ME, subjects were instructed to perform physical movement immediately upon cue appearance, and not before it. During the MI, subjects were instructed to perform motor imagery immediately upon cue appearance, and not before it. Tongue and eye movements were detectable through the EEG electrodes. Additionally, subject self-reports indicated that subjects were not performing ME or MI before cue appearance. A closed-circuit video was used to ensure that subjects performed ME or MI correctly and were awake and attentive. There was a 10 min rest period between every session in case of fatigue. Each subject was enrolled in the study for 2–2.5 h.

### 2.3. Trial Exclusion

During the experiment, according to the closed-circuit video, trials with wrong movements during ME were excluded; trials with any movements during MI were excluded. After the experiment, trials that contained muscle tension, tongue movement, face movement, eye movement, or eye blink artifacts in the 2 s preceding cue appearance and during cue were identified offline and excluded from all subsequent visualizations and analyses.

### 2.4. Feature Extraction

The raw EEG signal was filtered in an 8–30 Hz band. The frequency band was chosen because it includes the alpha and beta frequency bands, which have been shown to be most important for movement classification [20]. To show the ERD/ERS of each class (left hand movement, right hand movement and both feet movement), the EEG signals of C3, Cz and C4 channels were averaged online over the 3 s preceding and 5 s following cue appearance across all trials and all subjects. ERD/ERS is defined as the percentage of power decrease or power increase in relation to a reference period (in this study 0–1 s) [17], according to the expression

(1) ERD/ERS%=(A−R)/R×100%

The power within the frequency band of interest in the period after the event is given by *A*, whereas that of the reference period is given by *R*. ERD/ERS visualizations were created by generating time-frequency maps using a fast Fourier transformation (FFT) with Hanning windows, a 100 ms segment length, and no overlap between consecutive segments [32].

**Figure 4:**
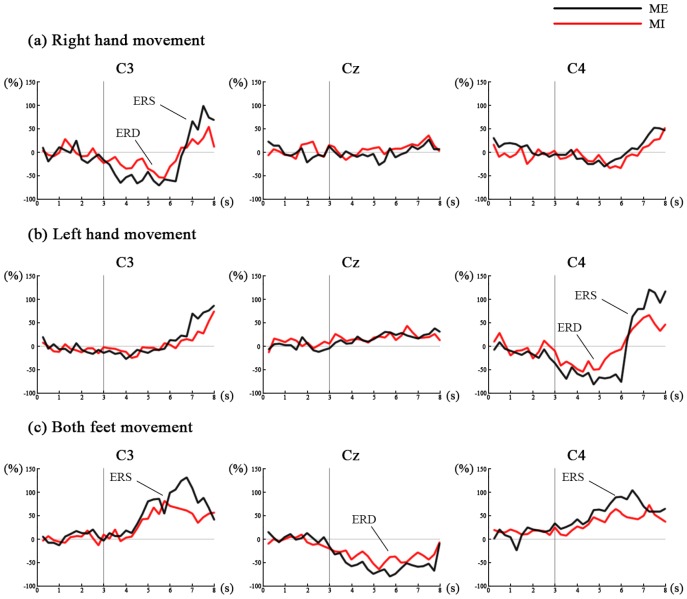
Time course of ERD/ERS. For each movement (right hand, left hand or both feet movement), the EEG power of C3, Cz and C4 within 8–12 Hz frequency band is averaged offline over the 3 s preceding and 5 s following cue appearance across all trials and all subjects, and is displayed relative (as percentage) to the power of the same EEG derivations recorded during the reference period: (**a**) shows the ERD/ERS patterns happening in the right hand movement; (**b**) shows the ERD/ERS patterns happening in the left hand movement; and (**c**) shows the ERD/ERS patterns happening in both feet movement.

**Figure 5:**
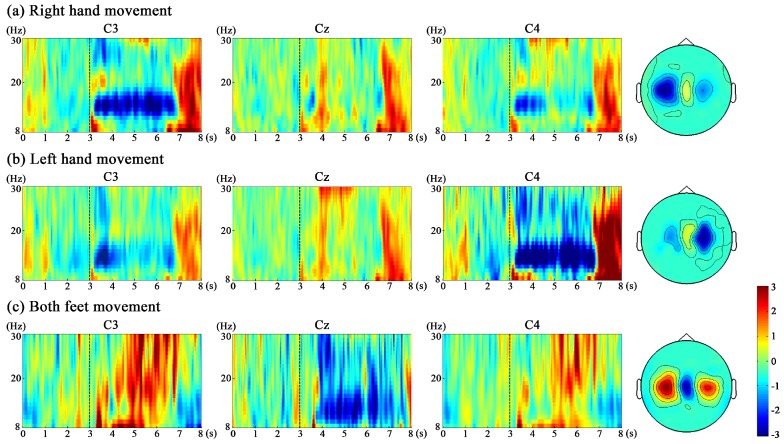
A representative example of time-frequency maps for ME of three movements from subject 3. (**a**) time-frequency maps of right hand movement; (**b**) time-frequency maps of left hand movement; (**c**) time-frequency maps of both feet movement. For each movement, time-frequency maps of channel C3 over the left sensorimotor cortex, C4 over the right sensorimotor cortex and Cz are illustrated. In the time-frequency map, 3 s means the cue occurrence. The **blue** color stands for ERD (power decrease), and the **red** color stands for ERS (power increase). The head topographies corresponding to the best frequency band (12–16 Hz for subject 3) from seconds 4–7 are provided next to the time-frequency maps.

According to the visual inspection of ERD and ERS (refer to the Figure 4 and Figure 5 in Results section), the time period 1–4 s after the cue appearance (seconds 4–7) and the best frequency band for each subject were selected in order to obtain the strongest ERD/ERS to extract features. Each selected time period was analyzed using time segments of 200 ms. Common spatial pattern (CSP) is widely used and highly successful in the binary case [33], which was applied in this study to perform feature extraction. Given two distributions in a high-dimensional space, the CSP algorithm can calculate spatial filters that maximize the variance between two classes [34]. For the analysis, let *V* denote the raw data of a single trial, an N×T matrix with *N* the number of channels (28 channels in this study) and *T* the number of samples in time. The normalized spatial covariance of the EEG can be calculated as

(2) $$W=\frac{VV^{T}}{trace(VV^{T})}$$

where ${}^{T}$ denotes the transpose operator and trace(a) is the sum of the diagonal elements of *a*. For each of the two distributions to be separated (i.e., left hand and right hand movement, or left hand movement and both feet movement), the spatial covariance $\bar{W}$ is calculated by averaging over the trials of each class. The composite spatial covariance is given as $W_{c}=\bar{W_{1}}+\bar{W_{2}}$. $W_{c}$ can be factored as $W_{c}=E_{c}\lambda_{c}E_{c}^{T}$, where $E_{c}$ is the matrix of eigenvectors and $\lambda_{c}$ is the diagonal matrix of eigenvalues. The whitening transformation $M=\sqrt{\lambda_{c}^{-1}}E_{c}^{T}$ equalizes the variances in the space spanned by $E_{c}$, i.e., all eigenvalues of $MW_{c}M^{T}$ are equal to one. If $\bar{W_{1}}$ and $\bar{W_{2}}$ are transformed as $P_{1}=M\bar{W_{1}}M^{T}$ and $P_{2}=M\bar{W_{2}}M^{T}$, then $P_{1}$ and $P_{2}$ share common eigenvectors *U*, i.e., if $P_{1}=U\lambda_{1}U^{T}$ and $P_{2}=U\lambda_{2}U^{T}$, then $\lambda_{1}+\lambda_{2}=I$, where *I* is the identity matrix. With the projection matrix $B={\left(U^{T}B\right)}^{T}$, the decomposition (mapping) of a trial *V* is given as Z=BV. The features we use for classification are obtained from the variances of the first and last *m* rows of the expansion coefficients $Z_{i}$, since, by construction, they are the best suited to distinguish between the two conditions. Let $D_{j}^{i}$ be the variance of the *j*-th row of $Z_{i}$, j=1,2…2m. The feature vector for trial *i* is composed of the 2m variances $D_{j}^{i}$, normalized by the total variance of the projections retained, and log-transformed as

(3) $$F_{j}^{i}=\log\left(\frac{D_{j}^{i}}{\sum_{j=1}^{2m}D_{j}^{i}}\right)$$

The feature vectors $F_{j}^{i}$ are fed into the classifier.

### 2.5. Classification

In the decoder-training phase, we applied linear discriminant analysis (LDA), support vector machine (SVM) and backpropagation neural network (BPNN) as the classifiers in this study because of their remarkable robustness and high performance in the BMI applications [35,36]. The input was the feature vector from CSP, and the output was one class (left hand movement, right hand movement or both feet movement). We separated the whole data set into the training set and testing set, and classified the data from the testing set with the model obtained from the training set. For testing the method optimally on the data available to us, we performed the 10-fold cross-validation procedure in the model training phase, i.e., nine folds of data of the training set were used for training and the remaining one fold of data was used for validation. Three different ratios between the training set and testing set were used, i.e., 50%–50%, 60%–40% and 80%–20%. Different classifiers and different train–test ratios can lead to different classification performance. Thus, we compared nine classification strategies in total (three classifiers × three train–test ratios) in order to select the best classifier and train–test ratios. Then, based on the best classification strategy, the better paradigm was selected. In the online-control phase, all data were used for testing data to test the classification models of the selected paradigm.

### 2.6. Data Analysis

To evaluate the proposed BMI performance, four measurements, i.e., accuracy, precision, recall and F-score, were used in this study. Accuracy is the ratio that the model correctly classifies the input. It can be calculated using the equation accuracy=(TruePositive+TrueNegative)/(TruePositive+TrueNegative+FalsePositive+FalseNegative). Precision is a measurement indicating the fraction that the model correctly predicts true class while recall indicates the fraction that the model detects true class. Precision and recall can be obtained using equations precision=TruePositive/(TruePositive+FalsePositive) and recall=TruePositive/(TruePositive+FalseNegative), respectively. F-score is an extended measurement of accuracy, and equally combines precision and recall. F-score can be computed using equation F−score=2recallprecision/(recall+precision). In the decoder-training phase, to evaluate the motor mode × paradigm interaction and motor mode and paradigm main effects, a 2 (ME or MI) × 2 (left versus right hand movement or left hand versus both feet movement) within-subjects ANOVA was applied. In the online-control phase, to evaluate the motor mode × scenario interaction and motor mode and scenario main effects, a 2 (ME or MI) × 2 (wearing or without wearing the exoskeleton) within-subjects ANOVA was applied. All statistics used were carried out at 95% confidence interval.

## 3. Results

After trial exclusion, all remaining data were processed and analyzed from the four subjects in the experiment.

### 3.1. Results of the Decoder-Training Phase

#### 3.1.1. Neurophysiological Analysis of ERD/ERS

To show the time course of ERD/ERS, for each movement (right hand, left hand or both feet movement), the EEG power of C3, Cz and C4 within the 8–12 Hz frequency band is averaged offline over the 3 s preceding and 5 s following cue appearance across all trials and all subjects, and is displayed relative (as percentage) to the power of the same EEG derivations recorded during the reference period, as shown in Figure 4. During the ME and MI of right hand and left hand (the time period 1–5 s after the cue appearance (seconds 4–8)), the EEG data reveals a significant ERD and post-movement ERS over the contralateral side, while only a weak ERS can be seen over the ipsilateral side and at the Cz electrode. During the ME and MI of both feet, the EEG data reveals a significant ERD at the Cz electrode, while a significant ERS at the C3 and C4 electrodes. Hand movement can result in a hand area (C3 and C4) ERD and simultaneously in a foot area (Cz) ERS, and foot movement can result in an opposite pattern. In addition, for all three movements, the ERD and ERS are more significant during ME than MI.

Figure 5 shows a representative example of time-frequency maps for ME of three movements from subject 3. For each movement, time-frequency maps of channel C3 over the left sensorimotor cortex, C4 over the right sensorimotor cortex and Cz are illustrated. In the time-frequency map, 3 s means the cue occurrence. The blue color stands for ERD (power decrease), and the red color stands for ERS (power increase). For the right hand and left hand movement, ERD is observed from around 1–4 s (seconds 4–7) after the cue onset due to the response delay; ERD in both alpha and beta bands is observed over motor areas contralateral to the hand moves. The ERS is mainly observed over around second 7–8 in the beta band over the contralateral motor areas. For the both feet movement, the ERD in both alpha and beta bands is observed at Cz electrode and the ERS in both alpha and beta bands is observed at the hand area (C3 and C4) from seconds 4–7. For subject 3, the time-frequency maps show that 12–16 Hz is the best frequency to obtain the strongest ERD/ERS to extract features. The head topographies corresponding to this frequency band from seconds 4–7 are provided next to the time-frequency maps. The best frequency bands for the other three subjects are: 8–12 Hz for subject 1, 18–22 Hz for subject 2, and 14–18 Hz for subject 4.

#### 3.1.2. Classification Results

**Figure 6:**
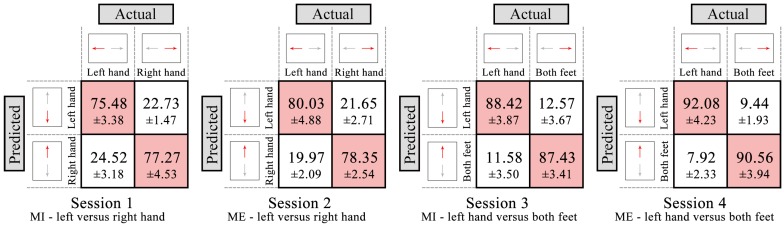
Confusion matrices (in %) for four sessions using S3 classification strategy in the decoder-training phase, averaged across all subjects. The numbers of each entry in the matrix represent the mean value and standard deviation across all subjects. The main diagonal entries stand for the correct classification, and the off-diagonal entries stand for the misclassification.

According to Figure 4 and Figure 5, the time period and the best frequency band for each subject were selected for feature extraction and classification. For each session, nine classification models based on different classification strategies (S1–S9) were trained to distinguish between two movements (left versus right hand movement, or left hand versus both feet movement). Table 2 shows classification accuracies of nine classification models for each session across all subjects. The LDA (S1–S3) and SVM (S4–S6) achieve the similar classification accuracies, which are higher than those of BPNN (S7–S9). For the different train–test ratios, the classification accuracies of 80%–20% (S3, S6 and S9) are higher than those of 50%–50% (S1, S4 and S7) and 60%–40% (S2, S5 and S8). S3 has a highest average classification accuracy of 83.71% ± 7.07%. These results indicate that using LDA classifier and 80%–20% train–test ratio (S3) achieves a better classification performance, which is further analysed to select the better paradigm. The confusion matrices of four sessions using S3 classification strategy are shown in Figure 6. The numbers of each entry in the matrix represent the mean value and standard deviation across all subjects. The main diagonal entries stand for the correct classification, and the off-diagonal entries stand for the misclassification. The four sessions achieve mean classification accuracy of 76.38% ± 4.25%, 79.19% ± 3.41%, 87.93% ± 4.83%, and 91.32% ± 2.89%, respectively. The accuracies of ME sessions (Sessions 2 and 4) are higher than those of MI sessions (Sessions 1 and 3); the accuracies of left hand versus both feet movement sessions (Sessions 3 and 4) are higher than those of left versus right hand movement sessions (Sessions 1 and 2). Table 3 shows mean precision, recall and F-score of the two classes of four sessions using S3 classification strategy across all subjects. In general, the results show high precision, recall and F-score indicating that the model has a high performance (Sessions 3 and 4). ANOVA shows no significant interaction between motor mode and paradigm (p>0.05), while there is a significant main effect of motor mode (*F* = 17.473, *p* = 0.004) and paradigm (*F* = 15.880, *p* = 0.016) for the classification accuracy. These results indicate that left hand versus both feet movement paradigm achieves a better classification performance, which would be used in the online-control phase.

### 3.2. Results of Online-Control Phase

**Figure 7:**
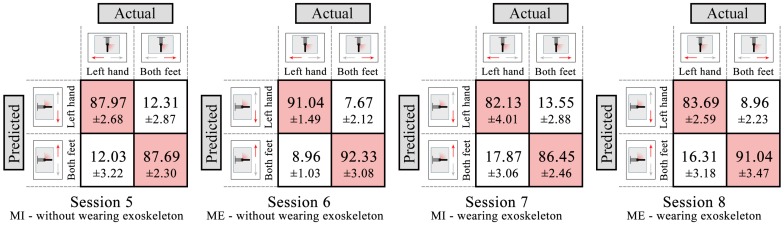
Confusion matrices (in %) for four sessions in the online-control phase, averaged across all subjects. The numbers of each entry in the matrix represent the mean value and standard deviation across all subjects. The main diagonal entries stand for the correct classification, and the off-diagonal entries stand for the misclassification.

The trained decoder using the selected paradigm was tested in the online-control phase. In two scenarios , subjects performed ME or MI to control the exoskeleton, resulting in four sessions (Session 5: MI, without wearing exoskeleton; Session 6: ME, without wearing exoskeleton; Session 7: MI, wearing exoskeleton; Session 8: ME, wearing exoskeleton). The trained model (LDA classifier) of Session 3 was used in Sessions 5 and 7; the trained model of Session 4 was used in Sessions 6 and 8. The confusion matrices of Sessions 5–8 are shown in Figure 7. The numbers of each entry in the matrix represent the mean value and standard deviation across all subjects. The main diagonal entries stand for the correct classification, and the off-diagonal entries stand for the misclassification. The four sessions achieve mean classification accuracy of 87.83% ± 2.79%, 91.69% ± 2.95%, 84.29% ± 2.11%, and 87.37% ± 3.06%, respectively. The accuracies of ME sessions (Sessions 6 and 8) are higher than MI sessions (Sessions 5 and 7); the accuracies of without wearing exoskeleton sessions (Sessions 5 and 6) are higher than wearing exoskeleton sessions (Sessions 7 and 8). Table 4 shows mean precision, recall and F-score of the two classes of Sessions 5–8 across all subjects. The results show the higher precision, recall and F-score of Sessions 5 and 6 than those of Sessions 7 an 8. ANOVA shows no significant interaction between motor mode and scenario (p>0.05), while there is a significant main effect of motor mode (*F* = 30.327, *p* < 0.001) and scenario (*F* = 24.502, *p* < 0.001) for the classification accuracy. These results indicate that without wearing exoskeleton scenario achieves a better classification performance than wearing exoskeleton scenario.

## 4. Discussion

By using physiological signals associated with natural behavior, users can interact with the environment through ME or MI. In this study, we proposed a BMI, and investigated whether self-induced variations of the EEG can be useful as control signals for an upper-limb exoskeleton.

In the decoder-training phase, subjects performed ME or MI under two paradigms, i.e., left versus right hand movement and left hand versus both feet movement. During the ME and MI of right hand and left hand, we observed contralateral ERD and ERS in the alpha and beta band during sustained movements and post-movement from all subjects, while only a weak ERS could be seen over the ipsilateral side and at the Cz electrode, as shown in Figure 4 and Figure 5. These facts are because there is a decrease in synchrony of the underlying neuronal populations during ME or MI (ERD), and ERS reflects a somatotypically specific, short-lived brain state associated with deactivation (inhibition) and/or resetting of motor cortex networks which happens in the post-movement [17,37]. In contrast to the hand movement, during the both feet movement, a significant ERD could be found close to electrode Cz overlying the primary foot representation area, while a significant ERS at the electrode C3 and C4 overlying the primary hand representation area, as shown in Figure 4 and Figure 5. These results give evidence of the existence of not only a “hand area mu rhythm” but also a “foot area mu rhythm” [38]. Each primary sensorimotor area may have its own intrinsic rhythm, which is blocked or desynchronized when the corresponding area becomes activated. Although the ERD/ERS patterns were expected to be similar between ME and MI, the amplitudes of ERD/ERS for MI sessions (Sessions 1 and 3) were smaller than those for ME sessions (Sessions 2 and 4). The reason for the fact that MI has less robust performance than ME might be that MI is not a natural behavior and thus requires more effort than ME [39]. In addition, compared with ME, there is no neural feedback in MI which may exhibit less activity (ERS) in the motor cortex and result in a lower signal to noise ratio. However, considering that MI is more meaningful for BMI application, more training should be done for the subjects with MI. Regarding the paradigm selection, the accuracies of left hand versus both feet movement paradigm (Sessions 3 and 4) are higher than those of left versus right hand movement paradigm (Sessions 1 and 2). These facts indicate that ERD and ERS patterns are highly differentiable between left hand and both feet movement, whereas they were less detectable between left hand and right hand movement. Therefore, we chose the left hand versus both feet movement paradigm to be used in the online-control phase.

In the online-control phase, the trained decoder using the selected paradigm was tested in two scenarios. The accuracies of ME sessions (Sessions 6 and 8) are higher than MI sessions (Sessions 5 and 7), which has the same results as those in the decoder-training phase. In addition, the accuracies of without wearing exoskeleton sessions (Sessions 5 and 6) are higher than wearing exoskeleton sessions (Sessions 7 and 8). One reason is that, when wearing the exoskeleton, the right arm will move following the exoskeleton, which causes some problems in practical use because the movement of the right arm would produce ERD/ERS patterns over the hand area and this can result in false decisions of the system [27], especially an effect on the classification of left hand movement. Even if the right arm movement is a passive movement, it can also result in the ERD/ERS patterns similar to those in voluntary movements [40]. A related work [41] about passive movement effect found that the ERD/ERS patterns associated with upper limb movements are not significantly changed by periodic lower limb passive movements, which is similar to our results (the right hand movement mainly affects the classification of left hand movement, but not foot movement, as shown in Figure 7). Therefore, one possible solution is that avoiding using ERD/ERS patterns over the hand area for feature extraction and classification in practical use, i.e., using the other paradigm like face versus foot movement, which mainly results in the ERD/ERS patterns over face area and foot area. This solution only applies to this study (upper-limb exoskeleton); we can still use ERD/ERS patterns over the hand area for the lower-limb exoskeleton or orthosis, but not the ERD/ERS patterns over the foot area.

Artifact contamination (tongue and eye movements, and EMG from facial muscles) in recording of EEG signal may possibly cause serious problems in BMI applications [40]. Throughout the experiment, tongue and eye movements, and EMG signals were monitored for all subjects, to make sure no artifacts during the ME and MI were performed. Furthermore, after the experiments, contaminated trials were identified online and excluded from all subsequent visualizations and analyses. Therefore, the artifact contamination was not a concern in this study.

The future work will cover the following two aspects: (1) the present results show that the proposed BMI are applicable to control an upper-limb exoskeleton for able-bodied subjects. However, BMI applications should also aim to the users affected by motor disabilities. Thus, we should apply the proposed method to these users to validate the results; and (2) in this paper, we aim to investigate whether self-induced variations of the EEG can be useful as control signals for an upper-limb exoskeleton. We explore how to classify binary movements. However, to control an exoskeleton, there are some other important points, like actuator control, control feedback, mechanical structure, etc., which need to be explored and studied.

## 5. Conclusions

In this paper, we investigated whether self-induced variations of the EEG can be useful as control signals for an upper-limb exoskeleton developed by us. A brain-machine interface based on ERD/ERS was proposed. In summary, ERD/ERS using left versus right hand movement paradigm and left hand versus both feet movement paradigm presented distinguishable patterns as we expected, both in MI and ME; self-induced variations of EEG can be useful as control signals for an upper-limb exoskeleton in two scenarios. Although the mean classification accuracy when subjects wore the exoskeleton was not so high as when subjects did not wear the exoskeleton during the online-control phase, the methods we proposed still exhibited satisfying properties and robust results. The present study demonstrates that the proposed BMI is effective to control the upper-limb exoskeleton, and provides a practical method by a non-invasive EEG signal associated with human natural behavior for clinical applications. It is worthwhile to pursue this potential system since BMI based on EEG can provide support in many ways for assisting daily activities and improving the quality of life of elderly, disabled and injured individuals.

## Figures and Tables

**Table 1:** The descriptions of four sessions in the decoder-training phase.

Session	Task	Paradigm	Cue and Description
Session 1	Motor imagery (MI)	left versus right hand movement	“←”: imagine left wrist extension;
			“→”: imagine right wrist extension.
Session 2	Motor execution (ME)	left versus right hand movement	“←”: execute left wrist extension;
			“→”: execute right wrist extension.
Session 3	Motor imagery (MI)	left hand versus both feet movement	“←”: imagine left wrist extension;
			“→”: imagine both feet dorsiflexion.
Session 4	Motor execution (ME)	left hand versus both feet movement	“←”: execute left wrist extension;
			“→”: execute both feet dorsiflexion.

**Table 2:** The classification accuracies of nine classification models based on different classification strategies for each session across all subjects (in %).

Classification Strategy	Classifier	Train-Test Ratio	Session 1	Session 2	Session 3	Session 4	Average
S1	LDA	50%–50%	74.34 ± 1.07	76.85 ± 2.22	85.38 ± 2.38	88.43 ± 3.41	81.25 ± 6.73
S2	LDA	60%–40%	75.44 ± 2.03	76.32 ± 1.93	87.88 ± 1.44	90.05 ± 2.54	82.42 ± 7.61
S3	LDA	80%–20%	76.38 ± 3.40	79.19 ± 2.83	87.93 ± 1.04	91.32 ± 3.01	83.71 ± 7.07
S4	SVM	50%–50%	75.03 ± 1.45	75.07 ± 4.05	85.55 ± 3.81	87.90 ± 1.56	80.89 ± 6.81
S5	SVM	60%–40%	75.81 ± 2.30	77.74 ± 2.12	86.47 ± 1.03	90.23 ± 2.44	82.56 ± 6.90
S6	SVM	80%–20%	76.55 ± 1.59	78.26 ± 3.13	87.47 ± 2.54	91.26 ± 1.23	83.39 ± 7.11
S7	BPNN	50%–50%	71.82 ± 2.39	73.11 ± 1.24	83.07 ± 3.09	86.34 ± 2,84	78.59 ± 7.21
S8	BPNN	60%–40%	72.13 ± 1.49	73.45 ± 1.83	83.23 ± 3.03	88.04 ± 2.57	79.21 ± 7.69
S9	BPNN	80%–20%	73.24 ± 2.73	75.33 ± 3.52	84.56 ± 1.91	88.43 ± 1.77	80.39 ± 7.27

S 1-9: Subject 1-9, LDA: linear discriminant analysis, SVM: support vector machine, BPNN: backpropagation neural network.

**Table 3:** The mean precision, recall and F-score of the different two classes of four sessions using S3 classification strategy in the decoder-training phase across all subjects (in %).

	Session 1 (MI)		Session 2 (ME)		Session 3 (MI)		Session 4 (ME)	
	Left Hand	Right Hand	Left Hand	Right Hand	Left Hand	Both Feet	Left Hand	Both Feet
Precision	76.86 ± 2.38	75.91 ± 0.92	78.71 ± 1.27	79.69 ± 2.36	87.55 ± 3.24	88.30 ± 2.03	90.70 ± 1.37	91.96 ± 2.07
Recall	75.48 ± 1.09	77.27 ± 1.33	80.03 ± 0.74	78.45 ± 1.04	88.42 ± 3.03	87.43 ± 1.92	92.08 ± 0.87	90.56 ± 1.90
F-score	76.16 ± 0.71	76.58 ± 0.31	79.36 ± 0.92	79.07 ± 0.48	87.99 ± 1.06	87.86 ± 0.77	91.38 ± 0.92	91.25 ± 0.88

MI: motor imagery, ME: motor execution.

**Table 4:** The mean precision, recall and F-score of the two classes of four sessions in the online-control phase across all subjects (in %).

	Session 5 (MI) without Exoskeleton		Session 6 (ME) without Exoskeleton		Session 7 (MI) wearing Exoskeleton		Session 8 (ME) wearing Exoskeleton	
	Left Hand	Both Feet	Left Hand	Both Feet	Left Hand	Both Feet	Left Hand	Both Feet
Precision	87.72 ± 1.09	87.94 ± 2.21	92.23 ± 1.17	91.15 ± 1.83	85.84 ± 1.37	83.96 ± 1.21	90.33 ± 1.16	84.81 ± 1.22
Recall	87.97 ± 0.93	87.69 ± 1.88	91.04 ± 1.23	92.33 ± 1.54	82.13 ± 1.26	86.45 ± 0.97	83.69 ± 1.46	91.04 ± 2.09
F-score	87.70 ± 0.35	87.81 ± 0.89	91.63 ± 0.43	91.74 ± 0.66	83.94 ± 0.88	85.19 ± 0.80	86.88 ± 0.45	87.81 ± 0.92

MI: motor imagery, ME: motor execution.

## Abbreviations

The following abbreviations are used in this manuscript:

BMI	brain-machine interfaces
EEG	electroencephalogram
ERD	event-related desynchronization
ERS	event-related synchronization
ME	motor execution
MI	motor imagery
ADL	activities of daily living
EMG	electromyography
LDA	linear discriminant analysis
CSP	common spatial pattern
